# Examining the Pro-Eating Disorders Community on Twitter Via the Hashtag #proana: Statistical Modeling Approach

**DOI:** 10.2196/24340

**Published:** 2021-07-09

**Authors:** Suku Sukunesan, Minh Huynh, Gemma Sharp

**Affiliations:** 1 Information Systems Deptartment Swinburne University of Technology Hawthorn Australia; 2 Department of Dietetics La Trobe University Melbourne Australia; 3 Monash Alfred Psychiatry Research Centre Monash University Melbourne Australia

**Keywords:** Twitter, infodemiology, eating disorders, proana, thinspo, hashtags, transient, cybersectarianism

## Abstract

**Background:**

There is increasing concern around communities that promote eating disorders (Pro-ED) on social media sites through messages and images that encourage dangerous weight control behaviors. These communities share group identity formed through interactions between members and can involve the exchange of “tips,” restrictive dieting plans, extreme exercise plans, and motivating imagery of thin bodies. Unlike Instagram, Facebook, or Tumblr, the absence of adequate policy to moderate Pro-ED content on Twitter presents a unique space for the Pro-ED community to freely communicate. While recent research has identified terms, themes, and common lexicon used within the Pro-ED online community, very few have been longitudinal. It is important to focus upon the engagement of Pro-ED online communities over time to further understand how members interact and stay connected, which is currently lacking.

**Objective:**

The purpose of this study was to explore beyond the common messages of Pro-ED on Twitter to understand how Pro-ED communities get traction over time by using the hashtag considered to symbolize the Pro-ED movement, #proana. Our focus was to collect longitudinal data to gain a further understanding of the engagement of Pro-ED communities on Twitter.

**Methods:**

Descriptive statistics were used to identify the preferred tweeting style of Twitter users (either as mentioning another user in a tweet or without) as well as their most frequently used hashtag, in addition to #proana. A series of Mann Whitney U tests were then conducted to compare preferred posting style across number of followed, followers, tweets, and favorites. This was followed by linear models using a forward step-wise approach that were applied for Pro-ED Twitter users to examine the factors associated with their number of followers.

**Results:**

This study reviewed 11,620 Pro-ED Twitter accounts that posted using the hashtag #proana between September 2015 and July 2018. These profiles then underwent a 2-step screening of inclusion and exclusion criteria to reach the final sample of 967 profiles. Over 90% (10,484/11,620) of the profiles were found to have less than 6 tweets within the 34-month period. Most of the users were identified as preferring a mentioning style of tweeting (718/967, 74.3%) over not mentioning (248/967, 25.7%). Further, #proana and #thinspo were used interchangeably to propagate shared themes, and there was a reciprocal effect between followers and the followed.

**Conclusions:**

Our analysis showed that the number of accounts followed and number of Pro-ED tweets posted were significant predictors for the number of followers a user has, compared to likes. Our results could potentially be useful to social media platforms to understand which features could help or otherwise curtail the spread of ED messages and activity. Our findings also show that Pro-ED communities are transient in nature, engaging in superficial discussion threads but resilient, emulating cybersectarian behavior.

## Introduction

The prevalence of eating disorders (EDs) has been on the rise ever since the condition was listed in the Global Burden of Disease Study [1]. Recent estimates show EDs claim the lives of 3.3 million people globally every year [2], a number that has doubled over the last 10 years [3]. Of all the ED types, anorexia nervosa (AN) in particular poses severe life-threatening health risks, with the highest mortality rate of all mental illnesses [4]. In addition, nonfatal presentations are listed as the fifth cause of chronic disease among adolescents aged 15-19 years old in the Australian female population [5].

Traditional media platforms and their representation of the “thin ideal” have long been associated with body dissatisfaction, a known risk and maintenance factor of EDs [6,7]. However, the media landscape has changed dramatically in recent years, and the last decade has seen a surge in social media use globally, where recent figures show more than half of the world’s population, or 3.8 billion people, are active on social media [8]. The use of the internet to communicate using common online platforms has become more popular due to the increasing focus on usability, the decreasing cost in access, and the ability of communications to cross large geographical distances [9]. This transition has seen the emergence of social and interpersonal support networks for users and in particular, the emergence of pro-eating disorder (Pro-ED) communities online [10-12].

Pro-ED communities are a controversial subculture that promotes positive attitudes toward EDs, namely AN (pro-anorexia/proana) and bulimia nervosa (pro-bulimia/promia). These communities share content to promote thinness, provide advice to other members, and glorify low body weight as ideal [13]. A shared group identity is formed through interactions between community members and can involve the exchange of “tips,” restrictive dieting plans, extreme exercise plans, and motivating imagery of thin bodies, also known as “thinspiration” or “thinspo” [11,14]. Boero and Pascoe [15] described these communities as being able to “bring people together who rarely talk about their disorder face to face in non-therapeutic settings” and noted that these groups are present online at their own will with no formal offline equivalent.

There is now a significant body of literature highlighting the way in which Pro-ED communities exist on the internet and in particular on social networking platforms such as Twitter [10,16,17]. The Twitter platform is a social networking service known for its microblogging capability and is used by 339.6 million people, mostly between the ages 18 years and 34 years [8]. Twitter users can create a profile known as a “handle” and post microblogs or “tweets,” typically text comprising 140 characters or less (although this was increased to 280 characters in 2017) from which other users can then share, known as a “retweet,” or follow other users to create their own personal, interconnected social network. The platform has become a center for online social activity and the quick exchange of information, with the option now to post content other than text such as images, videos, or web links, and users can contribute to larger conversations by adding keywords or hashtags within the tweet [10,16,17].

Hashtags can connect users and be used to form communities around common interest topics [18]. In the online Pro-ED community, #proana signifies a post supporting pro-ED attitudes and behaviors and is considered to be the established term to describe the Pro-ED movement’s consistent referencing of EDs (eg, explicit mentions of bulimia and AN) within these accounts. Most of these accounts have acquired followers who themselves posted about EDs [10]. Other research has focused on #thinspiration (“motivating” imagery of thin bodies) and #fitspiration (“motivating” imagery of “fit” bodies) and their use within a variety of social media formats. Across social media platforms, typically #thinspiration encourages more weight loss behaviors with a stronger connected community than that of #fitspiration [17]. However, both have been found to essentially share the same themes of encouraging guilt, dieting, and restraint [19-21]. Nevertheless, longitudinal hashtag research within the Pro-ED communities is still limited. Since Twitter does not currently have a policy for blocking such hashtags, unlike other social media sites such as Instagram, Facebook [22], and Tumblr [23], it presents a unique space to use these freely and has played host to a shift to a space in which the Pro-ED community now communicates [10,16].

Furthermore, research to date has typically focused on the characteristics of the specific social networking sites for interaction and overlooked the exploration of the broader implications of online communities. Indeed, a meta-analysis of pro-anorexia and pro-bulimia website studies reported that the main body of research has neglected the investigation of individual members that comprise the communities, including their behaviors, motivations, and state of health, instead examining the role and content of the websites in community building. However, research suggests that the effects of personal social groups and peer behavior are prominent features in this space. Allison et al [24] proposed that the forces of social imitation and competition drive group behavior and put forward the idea that the “authoritative voice” of AN partly results from the expectations of the social group. This finding was further supported by Ferguson et al [7], who suggested peer competition as more prominent than traditional media effects when looking at body dissatisfaction in teenage girls.

With this in mind, we suggest that focus needs to be placed upon the engagement of Pro-ED online communities to further understand how members interact, as Girvan and Newman [25] demonstrated through the creation of social graphs in which communication is visualized as relationships between entities. In addition, recent research suggests identifying terms, themes, and a common lexicon used within the Pro-ED online community as beneficial in understanding a Pro-ED identity [10,16]. Choudhury [26] looked at Tumblr to understand how both pro-anorexia and pro-recovery communities interact through tags and a common lexicon, with findings suggesting that AN content can be detected with a high level of accuracy due to distinctive “affective, social, cognitive, and linguistic style markers.” Chancellor et al [14] further explored the lexicon of Pro-ED community members, this time on Instagram, both before and after attempts of moderation by the social networking site to create a codebook of variations used to circumvent restrictions. A similar codebook of keywords was developed by Arseniev-Koehler et al [10] and Zhou et al [16] for Twitter, in an attempt to summarize and describe ED content. However, both of the studies did not track user profiles over time, and in particular, their study did not consider the tweeting styles of community members that could provide insights into how Pro-ED communities communicate and interact.

Our study sought to extend upon previous examinations of Pro-ED tweets and in particular examine profiles and engagement of Pro-ED communities together with preferred tweeting styles among Pro-ED users.

A secondary objective was to identify the most frequently used hashtag among Twitter profiles that include “proana” as a primary hashtag. A greater understanding of the Pro-ED communication networks on Twitter could have implications for the identification, prevention, and treatment of young people with EDs who may be receptive to online therapeutic interventions.

## Methods

### Ethics Approval

The current study was approved by the Swinburne University Human Research Ethics Committee (SUHREC) Project ID: 20190402-1922. In line with SUHREC advice, it was not possible to directly quote individual twitter usernames or their posts; thus, data are presented in aggregated form only.

### Sample

**Figure 1:**
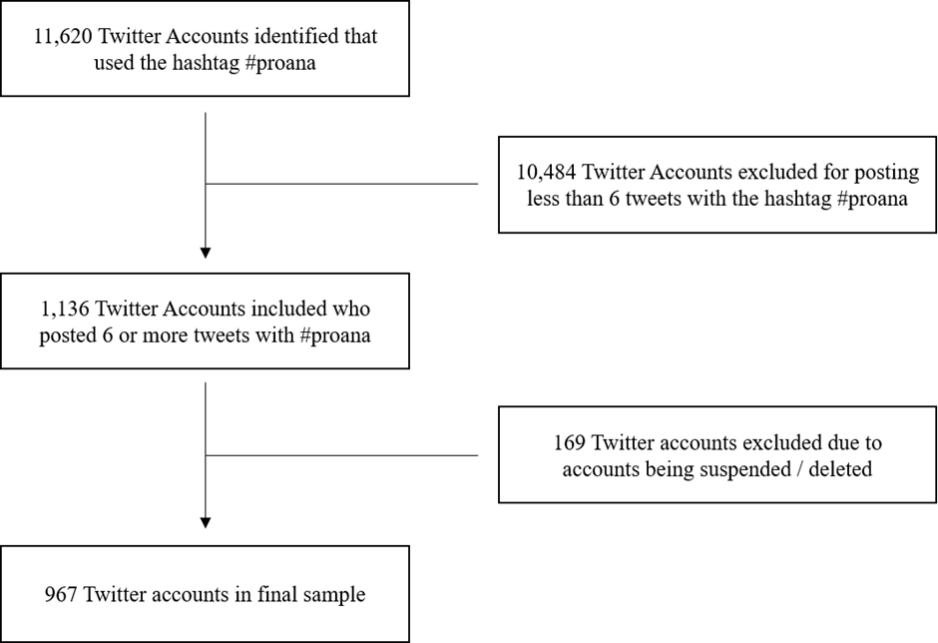
Selection of the sample for this study.

This study utilized publicly available Twitter data from Pro-ED profiles collected between September 15, 2015 and July 1, 2018, adhering to university ethics requirements. To identify Pro-ED profiles, we used an online scraping tool to gather posts (tweets) and reposts (retweets) using Twitter's public Application Program Interface (API). Twitter offers a systematic collection of sampled tweets as they are posted through a public API filtered by specific criteria. For this research, the hashtag #proana was the qualifying criteria, which resulted in 54,506 tweets and retweets (tweets that are recirculated by other users) across 11,620 Twitter profiles from various time zones and geographic locations. These profiles then underwent a double pseudonymization process to preserve anonymity before a 2-step screening process using inclusion and exclusion criteria was imposed to reach the final sample of 967 profiles (see Figure 1).

### Data Analysis

The 967 profiles were further classified into 1 of 2 categories (with-mention or without-mention) based upon the user’s preferred posting style. A without-mention message pertained to the user sending a tweet not to a particular individual, whereas a mention relates to the user including another Twitter account in the message. In the initial phase, descriptive statistics were ascertained to compare the proportion of tweets or retweets in relation to the user’s most frequently used hashtag. A series of Mann Whitney *U* tests were then conducted to compare preferred posting style across number of followed, followers, tweets, and favorites. Finally, a multiple linear regression model, using ordinary least squares [27], was then used to estimate the number of followers based upon the number of followed users, tweets, and favorites. The criteria for stepwise selection were based upon changes in the adjusted *R*^2^ values at each new step. Skewed predictor variables were trimmed by excluding extreme cases, as identified with a Cook distance >3 SDs from the mean. Analyses were conducted in SPSS Version 26.0 (IBM Corp, Armonk, NY).

## Results

The individual tweets or retweets across all 967 profiles were explored to ascertain the most frequently used hashtag associated with each account, as shown in Table 1. The “Top Hashtag” variable represents the most frequently used hashtag among users; for example, for this sample, 611 users (63.2%) had #thinspo as their most frequently used hashtag (excluding #proana). In contrast, the “Hashtag usage” variable relates to how many individual tweets across all accounts included the said hashtag.

**Table 1:** Comparing the top 10 most used hashtags (excluding proana) in this sample (967 users and 54,506 tweets or retweets).

Hashtag	Top hashtag, n (%)	Hashtag usage, n (%)
Thinspo	611 (63.2)	10,854 (20.53)
41DaysofStarvation	46 (4.8)	172 (0.33)
Ana	39 (4.0)	2615 (4.95)
Thinspiration	38 (4.9)	4499 (8.51)
Promia	31 (3.2)	2549 (4.82)
Anorexia	30 (3.1)	2537 (4.80)
Redbraceletpro	29 (3.0)	845 (1.60)
Skinny	21 (2.2)	2930 (5.54)
Bonespo	18 (1.9)	2429 (4.59)
ED book review	13 (1.3)	394 (0.75)
Total	876 (90.6)^a^	29,824 (56.42)^a^

^a^Only the top 10 hashtags are shown; thus, the % values do not equal 100.

The hashtag #41DaysOfStarvation was the most used hashtag for 46 users (46/967, 4.8%); this was the second highest category after #proana and #thinspo (Table 1). Conversely, #41DaysofStarvation was only mentioned in 172 (172/54,506, 0.33%) of the total tweets and retweets in this sample.

The 967 profiles were further classified into either “without-mention” or “with-mention” groups based upon their tweeting style. Overall, most profiles were classified as preferring “with-mention” tweeting styles (718/967, 74.3%) over “without-mention” tweeting styles (248/967, 25.7%). Table 2 displays the descriptive statistics for the 2 groups across the number of (1) profiles followed, (2) followers, (3) tweets, and (4) favorites. There were no significant differences between the 2 groups for all of the categories (followed, followers, tweets, and favorites).

**Table 2:** Online behaviors grouped by tweeting behavior.

Behaviors	Without mention (n=248)		With mention (n=718)		Mann-Whitney *U* test Z score	*P*
	Mean (SD)	Median	Mean (SD)	Median		
Followed	778.55 (2269.93)	168.5	758.23 (1938.82)	187.5	–0.88	.381
Followers	1001.16 (3605.59)	235.0	887.11 (2192.43)	210.0	–0.42	.675
Tweets	10390.46 (46922.71)	876.0	9886.84 (26378.60)	1281.0	–1.36	.173
Favorites	3476.15 (8781.05)	483.5	4228.01 (11702.89)	570.5	–0.74	.462

The relationships between the aforementioned factors (see Table 2) were also examined via a Pearson correlation test (see Table 3). The results indicated that all the factors were significantly correlated with each other, with the largest correlation being between number of followers and accounts followed.

**Table 3:** Intercorrelations of online behaviors (N=966).

Variable		Followed	Followers	Tweets
**Followers**				
	*r*	0.85	1	—^a^
	*P* value	<.001	—	—
**Tweets**				
	*r*	0.36	0.60	1
	*P* value	<.001	<.001	—
**Favorites**				
	*r*	0.30	0.30	0.42
	*P* value	<.001	<.001	<.001

^a^Not applicable.

The study further investigated if numbers of accounts followed, tweets, and favorites were significant predictors of number of followers. A forward stepwise model based upon the adjusted *R*^2^ was utilized to determine the best-fitting model:

*Followers* = –215.74 + 1.58(*Followed*) + 0.05(*Tweets*) **(1)**

In the final model (Table 4), favorites was no longer a significant predictor, with the remaining 2 predictors (followed and tweets) together explaining 35.5% of the variation in the number of followers.

**Table 4:** Regression coefficients for predictors of number of followers.

Predictor	Model 1^a^				Model 2^b^		
	B	SE	*P*	B		SE	*P*
Followed^c^	1.58	.10	<.001	1.58		.10	<.001
Tweets^c^	0.05	.01	<.001	0.05		<.01	<.001
Favorites^c^	<–0.01	.02	.558	N/A^d^		N/A	N/A

^a^Adjusted R^2^=0.354.

^b^Adjusted R^2^=0.355.

^c^Transformed.

^d^N/A: not applicable.

## Discussion

### Principal Findings

Our study explored the common lexicon of the Pro-ED Twitter community by identifying popular key words and phrases tagged in tweets. Results of this analysis indicate #thinspo as the most prominent hashtag within the Pro-ED Twitter community, other than #proana, suggesting considerable overlap between the topics and their intent. This indicates that wider conversation involving #thinspo across other social media platforms needs to be further scrutinized and treated as ED-related discussion. Previous research [17,19,28] has found that thinspiration tweeters, that is individuals using #thinspo or #thinspiration to accompany appearance- or weight-related posts on Twitter, form part of a closely connected genuine virtual community and differ to those propagating fitspiration content. Indeed, #fitspo or #fitspiration did not feature as one of our top 10 most used hashtags, suggesting that this hashtag is potentially identifying a different community focused more on the promotion of fitness and muscle building [28]. Taken together, these findings suggest #thinspo as a salient aspect of a Pro-ED lifestyle, with #proana and #thinspo used interchangeably in online spaces to communicate a supposedly motivating weight loss message to other community members.

Observing communication within online communities provides insight into their structure, member roles, and tribal behavior [29-31]. Typically, the communication patterns and network structures of online ED communities are differentiated by their intentional online behavior. Members of pro-recovery communities who view EDs as an illness and are actively working towards recovery generate more original content and actively seek out new profiles to follow when compared to Pro-ED communities [31]. Our findings suggest the communication patterns within Pro-ED Twitter communities to be more community driven. The type of tweet (with-mention vs without-mention) did not differ significantly across followed, followers, tweets, and favorites. This implies that whether a member is the source of the content or merely sharing it, they are equally likely to contribute to the growing Pro-ED community and its formation. As previously suggested by Wang et al [31], members within the Pro-ED Twitter community use the platform as a tool for community engagement and not typically as a means of communication per se as indicated by the number of retweets within our findings. This is a crucial finding as there is a greater role that social media platforms can play in addressing the communication. In essence, social media platforms could fill this void with tools that can facilitate communication and extend ED-related discussions with the ED community users. One approach would be to channel them to external sites, such as the National Butterfly Foundation (official organization for ED-related matters in Australia), mediated by a chatbot for cost efficiency.

The Pro-ED community chatter was dominated by retweets, by 75%, rather than genuine threads of communication. For example, the exchanges featuring “41DaysofStarvation” were a passing superficial topic in a particular subgroup of users that garnered quick interest and then discontinued. This could be due to the members’ transient [32-34] nature, which prohibits them from building longer-lasting discussion threads, with over 10,484 profiles only engaging in no more than 6 tweets. It is possible that this “41 days” of extreme weight control led to a deterioration in their physical health and subsequent inpatient admission. However, this pattern of communication could also be indicative of the network structure. Previous findings indicate that Pro-ED communities have a far-reaching online community [15] but low reciprocity rates of communication with other users through replies and mentions [31]. This alludes to the allegiance the members have towards the topic and care shown among members but rarely are there extended discussion threads [35]. Additionally, the possibility of users being barred for violating the rules of engagement, especially if their postings included suicidal and self-harm messages, may account for hashtag attrition rates. This was evident within this study where 14.9% (169/1136) of the Twitter users had their account suspended or deleted between September 15, 2015 and July 1, 2018 as shown in Figure 1. A further analysis on the remaining 967 profiles revealed that only 632 profiles are currently still active, showing a 34% attrition from July 1, 2018 to September 15, 2020. For example, one Twitter user was barred for 2 months by Twitter for posting adverse Pro-ED content. This resulted in the removal of all previous postings and interactions. This account holder has since resumed being online with the same Twitter handle continuing posting Pro-ED content, however less active. This incident was documented by the authors due to the longitudinal data collected from Twitter, a strength of our study.

The data also showed retweets of the original postings being still “alive” on Twitter despite the corrective action by the platform. This leads us to question the amount of ED-related messages that could be retweeted and commented upon long after an account has been deleted. Impact of these unhealthy messages could be everlasting to the society. This warrants further investigation but also highlights the complexity in removing postings beyond the immediate network of an individual where postings are transversed fluidly and randomly. Social media platforms will need to take heed of the fact that user content lives beyond the immediate layer of where a posting has been initially lodged and could be shared across different platforms. This could impact policy development for content removal and moderation to avoid similar incidents to the live streaming of a mass shooting in Christchurch, New Zealand via Facebook [36]. To best address these and other contextual issues, social media platforms need to work closely with external support organizations to adopt a best practice approach. In the context of ED, Twitter will need to soon adopt national ED bodies as safety partners [37] to continuously engage and be advised on matters relating to ED.

From our analysis, there appears to be a reciprocal effect between followers and the followed. This implies that the Pro-ED community is resilient [38] and gains traction, as more and more people may be influenced to be part of it. This emulates cybersectarian society behaviors [39], whereby niche sentiments appeal to only a select community of people who propagate information and are virtually enduring. While opinion leaders and influencers have been found to exist within online ED communities [31], dominating members are not typically apparent. Dominating members can exert constant enforcement or exhibit power that could encourage members to change their allegiance behavior or even abandon the community [35]. As indicated through the type of tweets and number of retweets, the Pro-ED community engages with content and propagates it, and while externally, the community may appear just as an avenue for individuals seeking social support, the focus is potentially more about aligning with the collective identity of the community. Both issues with identity and social roles have been noted as risk and maintenance factors of EDs [31]. Adverse health outcomes of these groups have been observed over time on social media platforms in their desire to become “thin,” hence the crucial need for an understanding of the community structure and development of innovative intervention methods. When faced with mediation, cybersectarian groups typically react impulsively to go incognito and reappear after a length of time or remain hidden forever. For the health and safety of the members of these groups, a more participatory treatment intervention would potentially generate better outcomes compared with an outright ban, as noted by Casilli et al [40].

Understanding the vitality of Pro-ED communities is relatively complex and is reliant on the emergence of health fads and the traction of passing themes. Here, social media platforms such as Twitter would need to play a proactive role in addressing these issues. For example, Twitter should directly communicate to the 632 active profiles reported in this study to reduce further ED-related discussion and minimize sharing of related content that has a negative impact, as reported by Tiggemann and Zaccardo [41]. While Twitter has already undertaken some action within the suicide and self-harm space [42], more would be expected to follow, as Boyd [43] noted that adolescent users frequently turn to social media platforms including Twitter as a coping mechanism to diffuse external pressures threatening their mental health. It would also be beneficial for this approach of analysis to be replicated on other social media platforms to observe whether Pro-ED communities behave in the same manner across platforms. Importantly, future research should address how hashtags and other message content can be utilized to identify and reach individuals who are struggling with EDs and provide them with much needed therapeutic interventions. However, a challenge for interventions is the rapidly changing lexicon of the community [44]. As our findings indicate, hashtags accompanying Pro-ED events such as #41DaysofStarvation were short lived; however, #proana persists as a consistent theme in an otherwise transient community, potentially providing an ideal starting point for intervention.

Our analysis showed that the number of accounts followed and number of Pro-ED tweets posted were significant predictors for the number of followers of a user compared to likes. Hence the “like” counter is an obsolete predictor for ED engagement and activity. This important finding could potentially be useful to social media platforms to understand which features could help or otherwise curtail the spread of ED messages. A recent report about Instagram’s decision to turn off the “like” counter [45] might be futile to curtail ED, though the number of likes has been reported to give some indication of support [46,47].

### Limitations

There are several limitations to the current study. First, some data may have been omitted in the data collection process owing to the free data access from the Twitter streaming API, which normally constitutes about 1% of the whole Twitter data stream [48]. However, as mentioned by Cavazos-Regh et al [28], the percentage of private Twitter accounts is very small, and Twitter accounts default to a public setting. Results might be more reflective if we had subscribed to Twitter premium API services [49] and targeted tweets from personal accounts, and a larger sample size would have made this study more generalizable across the board. Further, a suite of other ED-related hashtags described in [16] would have contributed to a larger data set. These factors will be considered in future to improve research outcomes.

### Conclusions

Notwithstanding these limitations, our study contributes to the emerging literature examining Pro-ED content on social media platforms by providing an understanding of the Pro-ED communities and also the engagement of these groups. Continued research is needed to understand how we might use these messages and group dynamics to provide intervention and support to people with EDs in need.

## Abbreviations

AN: anorexia nervosa
API: application program interface
ED: eating disorder
Pro-ED: promote eating disorders
SUHREC: Swinburne University Human Research Ethics Committee
